# Functional and structural diversity in GH62 α-L-arabinofuranosidases from the thermophilic fungus *Scytalidium thermophilum*

**DOI:** 10.1111/1751-7915.12168

**Published:** 2014-09-29

**Authors:** Amrit Pal Kaur, Boguslaw P Nocek, Xiaohui Xu, Michael J Lowden, Juan Francisco Leyva, Peter J Stogios, Hong Cui, Rosa Di Leo, Justin Powlowski, Adrian Tsang, Alexei Savchenko

**Affiliations:** 1 Department of Chemical Engineering and Applied Chemistry, University of TorontoON, M5S 3E5, Canada; 2 Structural Biology Center, Argonne National LaboratoryArgonne, IL, 60439, USA; 3 Centre for Structural and Functional Genomics, Concordia UniversityMontreal, QC, H4B 1R6, Canada; 4 Department of Chemistry and Biochemistry, Concordia UniversityMontreal, QC, H4B 1R6, Canada; 5 Department of Biology, Concordia UniversityMontreal, QC, H4B 1R6, Canada

## Abstract

The genome of the thermophilic fungus *Scytalidium thermophilum* (strain CBS 625.91) harbours a wide range of genes involved in carbohydrate degradation, including three genes, *abf62A*, *abf62B* and *abf62C*, predicted to encode glycoside hydrolase family 62 (GH62) enzymes. Transcriptome analysis showed that only *abf62A* and *abf62C* are actively expressed during growth on diverse substrates including straws from barley, alfalfa, triticale and canola. The *abf62A* and *abf62C* genes were expressed in *Escherichia coli* and the resulting recombinant proteins were characterized. Calcium-free crystal structures of Abf62C in apo and xylotriose bound forms were determined to 1.23 and 1.48 Å resolution respectively. Site-directed mutagenesis confirmed Asp55, Asp171 and Glu230 as catalytic triad residues, and revealed the critical role of non-catalytic residues Asp194, Trp229 and Tyr338 in positioning the scissile α-L-arabinofuranoside bond at the catalytic site. Further, the +2R substrate-binding site residues Tyr168 and Asn339, as well as the +2NR residue Tyr226, are involved in accommodating long-chain xylan polymers. Overall, our structural and functional analysis highlights characteristic differences between Abf62A and Abf62C, which represent divergent subgroups in the GH62 family.

## Introduction

Plant-derived lignocellulosic biomass represents a major renewable energy resource, as well as a source of raw materials for production of bio-based products (Carroll and Somerville, 2009). However, its conversion into biofuels, fibres and other industrially important biomaterials is hampered by its complex structure, which requires appropriate catalysts to extract its constituents for industrial uses. In natural environments, filamentous fungi achieve conversion of lignocellulotic biomass through secretion of a plethora of diverse carbohydrate and lignin-degrading enzymes. Genome sequencing efforts have revealed that each filamentous fungus harbours 100 to 300 glycoside hydrolase (GH) protein-encoding genes that often include multiple members within a family. However, the number of characterized fungal GH family enzymes is relatively small compared with the numbers of sequenced fungal GH family genes. To better understand the bewildering diversity of these enzymes and their roles in degradation of complex substrates, detailed characterization of their molecular function and specificity is needed.

Arabinoxylan is a major component of the hemicellulose fraction of grasses, and is especially abundant in the endosperm wall of dietary grains such as wheat, triticale and oats (Henry, 1985). It is a heteropolysaccharide and consists of a main chain of β-1,4 linked D-xylopyranosyl sugar units with randomly distributed L-arabinose substituents. The arabinose substituents are linked through either α-1,2- or α-1,3- glycosidic bonds to xylose. Some xylose units of xylan may carry additional substituents such as 4-O-methyl glucuronic acid, acetyl group or arabinose sugar esterified by coumaric or ferulic acids (de O Buanafina, 2009). These modifications in the xylan chain increase its complexity and can make it refractory to degradation.

Natural decomposition of arabinoxylan requires coordinated actions of endo-1,4-β-xylanases (EC 3.2.1.8), α-L-arabinofuranosidase (EC 3.2.1.55), α-glucuronidase (EC 3.2.1.139), acetyl (xylan) esterase (EC 3.1.1.72), ferulic acid esterase (EC 3.1.1.73) and β-xylosidase (EC 3.2.1.37) (de Vries *et al*., 2000; Sørensen *et al*., 2003). Combinations of such enzymes have been used to design ‘minimal’ enzyme cocktails for efficient arabinoxylan hydrolysis (Sørensen *et al*., 2007) for industrial applications. Among these, the α-L-arabinofuranosidases are the *exo*-acting enzymes that specifically remove L-arabinofuranose decorations from xylan or arabinan constituents of hemicellulose and are distributed in CAZy families GH3, GH43, GH51, GH54 and GH62 (www.CAZy.org).

Arabinoxylan arabinofuranohydrolases belonging to the GH62 family specifically remove α-1,2- or α-1,3- linked L-arabinofuranose decorations from xylan (Kellett *et al*., 1990). Characterized fungal GH62 arabinoxylan arabinofuranohydrolases that act on arabinoxylan include those from *Aspergillus niger* (Gielkens *et al*., 1997), *Aspergillus tubingensis* (Gielkens *et al*., 1997), *Penicillium chrysogenum* (Sakamoto *et al*., 2011), *Coprinopsis cinerea* (Hashimoto *et al*., 2011) and *Penicillium funiculosum* (De La Mare *et al*., 2013). However, other fungal GH62 hydrolases have also been reported to be active against branched arabinan and/or arabinogalactan, including enzymes from *Aspergillus sojae* (Kimura *et al*., 2000), *Ustilago maydis* and *Podospora anserina* (Siguier *et al*., 2014).

Based on the sequences listed at CAZy, the GH62 family is proposed to consist of two distinct subfamilies (Hashimoto *et al*., 2011; Siguier *et al*., 2014). Many sequenced fungal genomes, such as those of *P. funiculosum* (De La Mare *et al*., 2013) and *Coprinopsis cinerea* (Hashimoto *et al*., 2011), have been reported to carry at least two or more GH62 hydrolases which may either belong to the same or different subfamilies. Recently, we sequenced the genome of *Scytalidium thermophilum* (http://fungalgenomics.ca/), a thermophilic ascomycete with optimum growth temperatures nearing 50°C. This fungus is the dominant organism of mushroom compost (Wiegant, 1992; Straatsma *et al*., 1994) and is a source of thermostable enzymes (Guimarães *et al*., 2001; Zanoelo *et al*., 2004) with possible commercial applications. In this work, we have characterized the GH62 hydrolases from this fungus in terms of their induction patterns on biomass substrates, structure, biochemical properties and structure–function relationships.

## Results

### Sequence analysis and expression of *S. thermophilum* GH62 hydrolases

**Fig 1:**
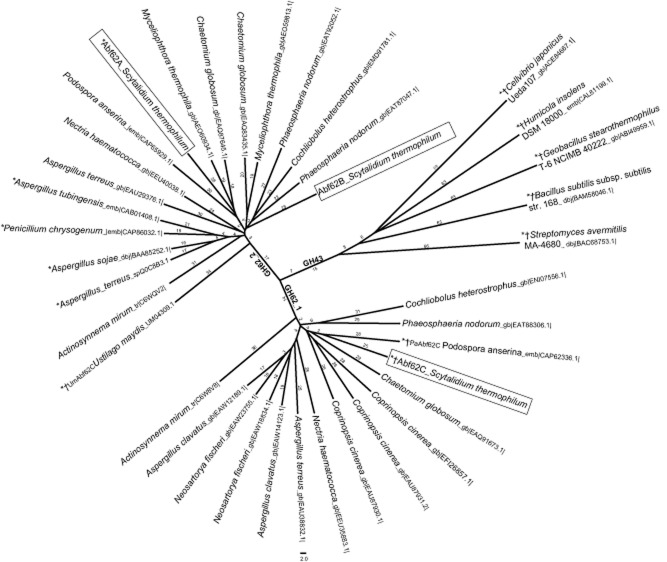
Phylogenetic distribution of fungal GH62 sequences into two subfamilies. A cladogram displaying branching of various fungal GH62 sequences into two subfamilies, GH62_1 and GH62_2, rooted at an out-group branch consisting of sequences from five well-characterized GH43 enzymes. The cladogram was calculated using neighbour-joining clustering methods of ClustalW2 and visualized using Figtree. The biochemically characterized enzymes are marked with an asterisk (*), and those with available structures are marked with symbol ‘‡’.

The genome sequence of the thermophilic fungus, *S. thermophilum* strain CBS 625.91 contains three genes – *abf62A*, *abf62B* and *abf62C* – predicted to encode secreted GH62 family arabinofuranosidases (Genbank accession numbers KJ545572, KJ545573 and KJ545574). Abf62A and Abf62B share 60% sequence identity between themselves, but only 34% and 36% sequence identity, respectively, with Abf62C (Table S1). A cladogram was constructed using GH62 sequences (Fig. 1) from various fungal genomes including *S. thermophilum* and was rooted at an out-group branch consisting of five distinct functionally and structurally characterized GH43 sequences: Arb43a from *Cellvibrio japonicas* [Protein Data Bank (PDB) i.d. 1GYD, (Nurizzo *et al*., 2002)], AbnB from *Geobacillus stearothermophilus* (2EXH, Brüx *et al*., 2006), BsAXH-m2,3, *Bacillus subtilis* subsp. subtilis (3C7E, Vandermarliere *et al*., 2009), SaAraf43A from *Streptomyces avermitilis* (3AKF, Fujimoto *et al*., 2010) and AXHd3 from *Humicola insolens* (3ZXJ, McKee *et al*., 2012). Notably, despite significant variation in substrate specificities, the five representatives of GH43 enzyme cluster as one out-group, whereas the fungal GH62 enzymes are distributed into two phylogenetically distinct subfamilies i.e. GH62_1 and GH62_2 (Hashimoto *et al*., 2011; Siguier *et al*., 2014). For the GH62 enzymes of *S. thermophilum* sequences of Abf62A/Abf62B belongs to subfamily GH62_2, while Abf62C is a member of the GH62_1 subfamily.

It is not uncommon for fungal genomes to harbour more than one GH62 gene, and some such as *C. cinerea* (Stajich *et al*., 2010) and *Myceliophthora thermophila* (Berka *et al*., 2011) may feature multiple representatives of the same subfamily (Fig. 1). The existence of two different subfamilies and multiple members of the same subfamily suggests the possibility of functional diversity among various GH62 homologues that favours their coexistence in the course of evolution.

The sequences of all three *S. thermophilum* GH62 enzymes feature an N-terminal signal motif typical of extracellular fungal proteins. Abf62A is the only one of the three enzymes that includes a motif, at the C-terminal, similar to carbohydrate-binding module 1 (CBM-1) in addition to the core catalytic domain. The cellulose-binding properties of CBM-1-containing GH62 enzymes from *C. cinerea* and *P. funiculosum* have previously been reported (Hashimoto *et al*., 2011; De La Mare *et al*., 2013).

**Fig 2:**
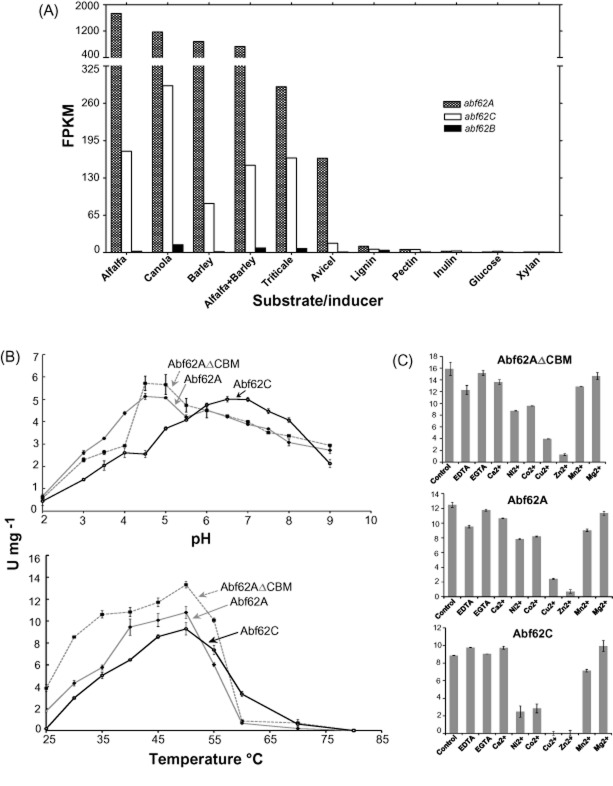
Transcription and biochemical analyses of three GH62 enzymes of *S. thermophilum.*A. RNA-Seq reads of gene transcripts of *abf62A*, *abf62C* and *abf62B* in *S. thermophilum* after growth on various complex substrates, as described in ‘Experimental procedures’.B. Determination of optimum reaction pH and temperature for Abf62C, Abf62A and Abf62AΔCBM using wheat arabinoxylan as substrate with reaction conditions as described in supplemental Appendix S1 Experimental procedures.C. Effect of divalent cation chelation (EDTA and EGTA, 2 mM each) and supplementation (Ca^2+^, Co^2+^, Mg^2+^, Mn^2+^, Ni^2+^, Cu^2+^ and Zn^2+^; 2 mM chloride salt of each) on enzymatic activities of Abf62C, Abf62A and Abf62AΔCBM using wheat arabinoxylan as substrate.

To probe the roles of *abf62A*, *abf62B* and *abf62C* in the degradation of different biomass substrates, *S. thermophilum* cultures were grown in media supplemented with various polysaccharides, lignin, straws or wood pulps as carbon source (Berka *et al*., 2011). The expression of individual GH62 members was quantified by transcriptome analysis using ribonucleic acid sequencing (Fig. 2A). Robust expression of *abf62A* and *abf62C* was observed in *S. thermophilum* cultures grown on complex substrates such as straws from alfalfa, canola, barley and triticale, while only basal or no expression was detected for *abf62B*. The expression of *abf62A* was generally higher than that of *abf62C*, reaching up to fivefold higher level in culture on barley straw. Since expression of *abf62B* was minimal during growth on any of the selected substrates, we focused on functional and structural characterization of Abf62A and Abf62C.

### Catalytic properties of *Abf62A* and *Abf62C*

For structural and functional characterization, Abf62A and Abf62C were produced in recombinant form in *Escherichia coli* and purified to homogeneity. DNA sequences encoding N-terminal signal peptides, corresponding to residues 1–30 of Abf62A and 1–18 of Abf62C were omitted during cloning, and proteins were produced with N-terminal polyhistidine tags. In addition, an Abf62A fragment designated Abf62AΔCBM that corresponds to the enzyme's core catalytic domain (residues 30 to 322) was also produced.

Recombinant proteins were purified to homogeneity and tested for activity using pNP-α-L-arabinofuranoside. Since Abf62A, Abf62AΔCBM and Abf62C were all active against this generic substrate, activity was next tested against wheat arabinoxylan (a linear xylan backbone with α-1,2 and α-1,3 linked L-arabinofuranose), as well as branched sugar beet arabinan (which has terminal α-1,2 and α-1,3 L-arabinofuranose on an α-1,5-L-arabinofuranose linked main chain) (Table 1). Abf62A and Abf62C are both active against these substrates but have no detectable activity against CM-linear 1,5-α-L-arabinan, which contains exclusively non-terminal 1,5-α-L-arabinofuranose linkages, indicating that these enzymes lack *endo*-arabinanase activity. Since the enzymes are active against pNP-α-L-arabinofuranoside and branched arabinan as well as arabinoxylan, we refer to them as arabinofuranosidases rather than arabinoxylan arabinofuranohydrolases (Kormelink *et al*., 1991).

**Table 1:** Kinetic parameters of *Abf62C* and *Abf62A* wild type and variants on arabinan substrates

Protein	High-viscosity wheat arabinoxylan		pNP-α-L-arabinofuranoside	Sugar beet arabinan
	K_m_	k_cat_	Specific activity	Specific activity
	mg ml^−1^	min^−1^	U mg^−1^	U mg^−1^
**Abf62C**	3.66 ± 0.82	16.55 ± 1.13	0.02	1.5 ± 0.38
Abf62C_Y107A	7.56 ± 0.69	14.88 ± 0.62	0.01	N.A.
Abf62C_Y168A	22.53 ± 1.59	38.49 ± 2.27	0.01	N.A.
Abf62C_Y168T	6.02 ± 0.24	14.81 ± 2.44	N.D.	N.A.
Abf62C_Y226A	0.54 ± 0.29	3.67 ± 0.23	N.D.	N.A.
Abf62C_D194A	N.D.	N.D.	0.38	N.A.
Abf62C_W230A	N.D.	N.D.	0.35	N.A.
**Abf62A**	9.38 ± 1.98	51.44 ± 5.02	0.24	2.8 ± 0.65
**Abf62A-ΔCBM**	8.40 ± 2.02	67.09 ± 7.14	0.86	7.3 ± 0.28

N.A., not analysed; N.D., no activity detected.

Abf62A and Abf62C showed differences in relative activities on different substrates, as well as in kinetic parameters (Table 1, Fig. S1A). The k_cat_ and K_m_ determined for Abf62A on wheat arabinoxylan are both threefold higher than those determined for Abf62C. The specific activity on pNP-α-L-arabinofuranoside is about 10-fold higher for Abf62A versus Abf62C, and for sugar beet arabinan the specific activity of Abf62A is twice that of Abf62C. The activities of the Abf62AΔCBM fragment are consistently higher than those of the full-length enzyme. The temperature and pH optima for Abf62A and Abf62C were obtained using reducing sugar assays with wheat arabinoxylan as substrate (Fig. 2B). The enzymes are optimally active at 50°C and at pH ranges of 5.0–6.5 (Abf62A) and pH 5.5–7.0 (Abf62C). ^1^HNMR spectroscopy showed that both Abf62C and Abf62A are active against both α-1,2 and α-1,3 L-arabinofuranosyl linkages in wheat arabinoxylan (Fig. S1B). Further, Abf62C generated arabinose as the sole end product from wheat arabinoxylan as confirmed by Dionex chromatography (Fig. S1C).

Previous studies of GH43 family enzymes showed that their activities were increased significantly in the presence of various divalent cations (Jordan *et al*., 2013; Lee *et al*., 2013; Santos *et al*., 2014) such as Ca^2+^, Co^2+^, Fe^2+^, Mg^2+^, Mn^2+^, Mn^2+^ and Ni^2+^, and inhibited by chelating agents (de Sanctis *et al*., 2010) or in the presence of Cu^2+^ or Zn^2+^ (Lee *et al*., 2013). The effects of divalent metal ions and chelators on Abf62A, Abf62ΔCBM and Abf62C activities on wheat arabinoxylan are shown in Fig. 2C. The presence of chelating agents such as EDTA (ethylene diamine tetraacetic acid) or EGTA (ethylene glycol tetraacetic acid) had little (< 10%) or no effect on the biochemical activity of Abf62C, whereas the activity of Abf62A was decreased by 20% in the presence of EDTA. Similarly, the presence of Ca^2+^ or Mg^2+^ resulted in only small changes (< 10%) in the activities of the two enzymes, whereas the presence of Ni^2+^, Co^2+^, Zn^2+^, Cu^2+^ or Mn^2+^ inhibited both enzymes in accordance with the order of their atomic radii Zn^2+^ > Cu^2+^ > Co^2+^ > Ni^2+^ > Mn^2+^. The degree of inhibition was somewhat greater for Abf62C compared with Abf62A.

To summarize, both Abf62A and Abf62C were active on the same set of substrates tested, but the Abf62A enzyme exhibited significantly higher specific activities than Abf62C. These enzymes also showed small variations in their optimal pH range and relative sensitivities to divalent cations.

### Structural characterization of *Abf62C*

The crystal structures of Abf62C in apo form and in complex with xylotriose were determined to 1.23 Å and 1.48 Å resolutions respectively. The Abf62C apo structure was determined using a selenomethionine substituted protein crystal by the single wavelength anomalous diffraction (SAD) method (Hendrickson, 1991), and it was then used as a search model to determine the structure of the Abf62C-xylotriose complex by the molecular replacement method (Vagin and Teplyakov, 1999). The statistics for both structures are presented in Table 2.

**Table 2:** Data and refinement statistics

Data collection statistics	*Apo-*Abf62C	*Xylotriose-*Abf62C
PDB ID	**4PVA**	**4PVI**
Space group	P3_1_	P3_1_
Unit cell (Å)	a = 72.0,	a = 49.6,
	b = 72.0,	b = 49.6,
	c = 61.2,	c = 232.6,
	α = β = 90°, γ = 120°	α = β = 90°, γ = 120°
Resolution (Å)	27.5–1.23	23.7–1.48
Wavelength (Å)	0.9794 Å	0.9794 Å
Number of observed reflections	461 208	112 129
Number of unique reflections	97 754	58 733
Redundancy^b^	4.7 (1.8)	1.9 (1.6)
*R*_merge_^a^^b^ (%)	4.4 (41.8)	2.5 (34.5)
*R_rim_*^a^^b^ (%)	4.9 (56.4)	3.3 (46.6)
Completeness^b^ (%)	94.7 (52.2)	96.5 (96.4)
I/σ	40.7 (1.80)	22.4 (1.90)
**Phasing**		
phasing method	SAD	MR
Refinement statistics		
*R*_cryst_ (%)	14.40	13.02
*R*_free_ (%)	16.21	15.88
protein residues	320	320
xylotriose	0	1
solvent	515	561
**RMSD from target values**		
bond lengths (Å)	0.013	0.011
bond angles (deg)	1.90	1.51
Average *B* factors (Å^2^)		
protein	16.39	16.09
Xylotriose	–	11.36
H_2_O	31.34	30.85
Ramachandran (%)^c^ M.F./A.A./O	**96.7/3.3/0**	96.7/3.0/0.3

a *R*_merge_ = Σ*_hkl_*Σ*_i_*|*Ii*_(_*hkl*_)_ − 〈*I_hkl_*〉|/Σ*_hkl_*Σ*_i_I_i_*_(_*_hkl_*_)_, where I*i*_(_*hkl*_)_ is the *i*th observation of reflection *hkl*, and 〈*I_hkl_*〉 is the weighted average intensity for all observations *i* of reflection *hkl*. 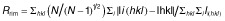, and 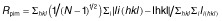.

b Numbers in parentheses are values for the highest-resolution bin.

c As defined by molprobity (M.F. –the most favored/A.A additionally allowed/O. outlier).RMSD, root mean square deviation.

**Fig 3:**
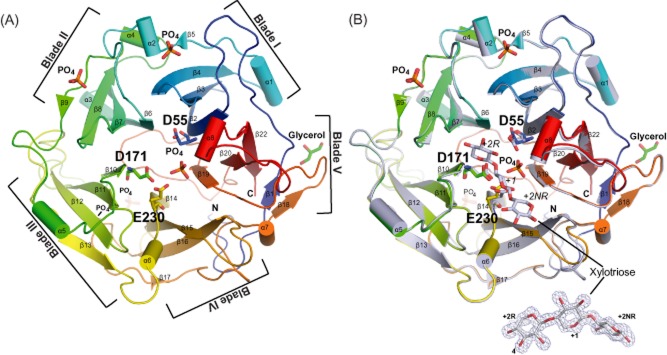
Crystal structure of Abf62C.A. Cartoon representation of the apo structure of Abf62C (in rainbow). The catalytic triad (Asp55, Asp171 and Glu230) residues, five co-crystallized PO_4_^2-^ ions and one glycerol molecule are displayed in sticks.B. Apo structure of Abf62C (in rainbow) is superimposed with xylotriose-bound Abf62C (grey). Xylotriose, the catalytic triad and the central PO_4_^2-^ molecule located in the active site are shown in sticks. A close-up view of the xylotriose molecule showing an excellent 2Fo-Fc density maps countered at 1σ (light blue).

The Abf62C apo structure contains one polypeptide chain (residues 30 to 350) in the asymmetric unit. In addition, five phosphate ions and one glycerol molecule were modelled (Fig. 3A). The overall fold of Abf62C adopts the five-bladed β-propeller fold similar to the other representatives of the GH43_62_32_68 superfamily (PDB id 2EXH, Brüx *et al*., 2006; PDB id 4N2R, Siguier, *et al*., 2014; PDB id 1WMY, Maehara, *et al*., 2014). Each ‘blade’ in this fold consists of either four (Fig. 3A, ‘blade’ I, II, IV and III) or five (Fig. 3A, ‘blade’ V) β-strands forming antiparallel β-sheets that are interconnected through loops of variable lengths to form a funnel-like structure that encircles the central cavity, which houses the active site.

A comparative sequence analysis was undertaken (Fig. S2) to detect sequence conservation among various members of the two GH62 subfamilies, including structurally characterized representatives from the basidiomycete *Ustilago maydis* (UmAbf62A) and the ascomycete *Podospora anserina* (Siguier *et al*., 2014) points to three completely conserved residues Asp55, Asp171 and Glu230 as the catalytic triad of Abf62C. The disposition of these catalytic residues in the Abf62C structure (Fig. 3A and B) is similar to that of catalytic triads previously characterized in GH43 (Brüx *et al*., 2006) and GH62 (Siguier *et al*., 2014) enzyme family representatives. Interestingly, one of the phosphate molecules present in the Abf62C apo structure is bound in the active site cavity forming a network of hydrogen bonds with side chains of active site residues (Lys54, Arg259, His303 and Gln328) including that of the catalytic Glu230, thus suggesting the probable position of arabinose binding (Fig. S3A and B).

**Fig 4:**
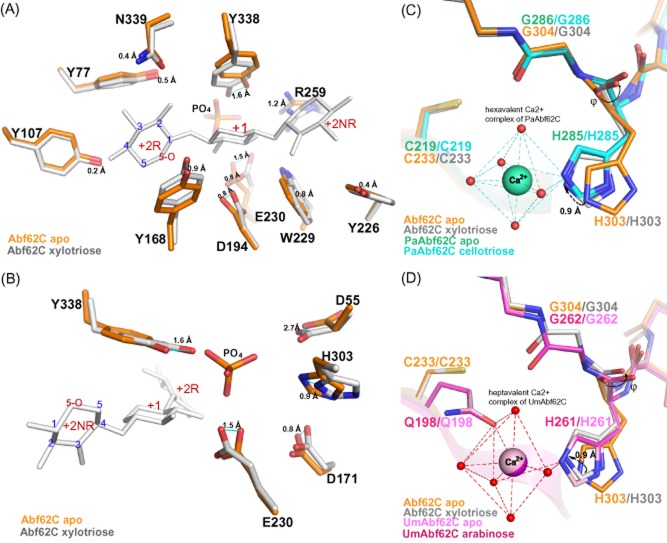
Conformational changes upon xylotriose and calcium ion binding.A. The relative shift of the xylostriose-interacting residues due to substrate binding towards molecular surface. The xylotriose-bound Abf62C residues (grey) are overlaid over apo-Abf62C residues (orange), and the xylotriose molecule is shown in white.B. Changes in the side chain conformations of the active site residues in the catalytic core.C. Comparative calcium ion binding between the *Pa*Abf62C and Abf62C apo and ligand bound structures.D. Comparative calcium ion binding between the *Um*Abf62C and Abf62C apo and ligand bound structures.

The Abf62C apo and xylotriose-bound structures superimposed with root-mean-square deviation of 0.24 Å over 324 C-alpha atoms (Fig. 3B), indicating minimal change in conformation upon substrate binding. However, detailed comparison of the apo and the xylotriose-bound Abf62C structures revealed the change in positions side chains of several residues (Fig. 4A and B) in response to binding to xylotriose, both towards the molecular surface (Tyr168, Trp229, Arg259 and Tyr338; Fig. 4A) and the catalytic core (Asp55, Asp171, Glu230 and His303; Fig. 4B). The most dramatic shifts were observed in Asp55 (2.7 Å and 2.2 Å of OD2 and OD1 atoms respectively), Tyr338 (1.7 Å shift of –OH group) and Glu230 (1.5 Å shift of atom OE2).

The recently characterized GH62 enzyme structures (Maehara *et al*., 2014; Siguier *et al*., 2014) contain a single calcium ion in the active site; a feature shared with previously characterized members of GH43 family as well (de Sanctis *et al*., 2010; Santos *et al*., 2014). Surprisingly, despite significant sequence similarity between *Pa*Abf62C and Abf62C enzymes (64% identity, Table S1) and also the conserved calcium binding residue (H285 in PaAbf62C and His303 in Abf62C; Fig. 4C), the Abf62C structure does not harbour a calcium ion in its active site. This Abf62C feature is in line with the functional data presented above, which shows that Abf62C activity is not significantly affected by the presence of Ca^2+^ or chelating agents, suggesting structural and functional independence from divalent metal ion binding.

However, a 0.9 Å shift of the ND1 atom of His303 in Abf62C (Fig. 4C and D) in the xylotriose bound structure places this residue in a virtually identical position to the calcium-coordinating orientation observed in the structures of the previously characterized enzyme *Pa*Abf62C (Fig. 4C) and *Um*Abf62C (Fig. 4D). The presence of a calcium ion in these two enzymes plays a structural role by restricting the movement of the histidine side chain. Apparently, substrate binding in Abf62C induces a conformational change of His303 from the preferred region in the apo structure to an energetically disallowed region of the Ramachandran plot for Abf62C-xylotriose (Fig. 4C and D).

### Xylotriose binding sub-site

**Fig 5:**
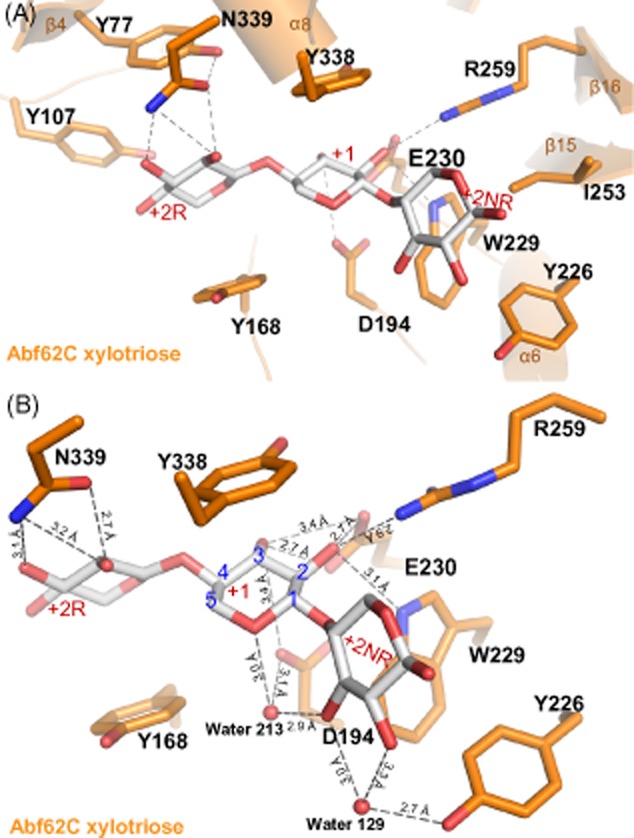
Binding topology of xylotriose in the substrate sub-cavity of Abf62C.A. The residues interacting with xylotriose (Asp194, Tyr168, Trp229, Glu230, Tyr226, Tyr338 and Asn339) and the pocket (Tyr77, Tyr107 and Ile253) are shown in orange and xylotriose is shown in silver. Hydrogen bonds are represented by dashes.B. Close-up view of the active site showing hydrogen-bond network (grey dash lines) formed between the xylotriose molecule (grey) and interacting residues and solvent (two water molecules).

The xylotriose molecule occupies in a curved cylindrical sub-cavity (Fig. S4A and B) leading into the active site and is constrained by helix α8, (residue Tyr338, blade I), helix α6 (Tyr226 ‘blade’ IV), the loop connecting helix α5 with strand β11 (Tyr168, connects ‘blades’ III and IV), loop between α-2 and stand β-6 (Tyr107, ‘blade II’). The binding cavity involves many polar residues that are visualized in a long patch of acidic residues in an electrostatic representation (Fig. S3B). To define the structural basis for substrate recognition by Abf62C, the substrate binding cavity was divided into three sub-sites (Fig. 5A and B) using the xylotriose sugar ring nomenclature (McKee *et al*., 2012). Thus, sub-site +2R is where the reducing end of the xylotriose backbone binds, placing the scissile bond containing the central xylose ring at sub-site +1 (the active site) and the xylose at non-reducing end binds at sub-site +2NR. The type of interactions and H-bonding distances are listed in Table S3. The bound orientation of xylotriose at the Abf62C active site positions the +1 xylose ring relative to the catalytic triad so that its two hydroxyl groups (C2 and C3) are within 3 Å distance of the general base residue, Glu230. These observations support the functional data showing that Abf62C is active against both α-1,2 and α-1,3 L-arabinofuranosyl linkages.

### Probing the *Abf62C* active site residues by mutagenesis

To test the individual roles of active site residues in substrate recognition and catalysis, corresponding Abf62C residues were individually substituted by alanine or other residues using site-directed mutagenesis (Table 1 and Table S4). The resulting variants were purified and tested for activity against wheat arabinoxylan and pNP-α-L-arabinofuranoside for comparison to the wild-type enzyme.

As expected, replacement of the catalytic triad (D55A, D171A and E230A) and surrounding core residues such as Lys54, Tyr77, Arg259, Tyr338 and His303 by alanine renders Abf62C completely inactive on both substrates (Table S4). Many of these residues form hydrogen bonds with the phosphate ion trapped in the active site of the Abf62C apo structure (Fig. S3A and B) and suggest their equivalent participation in L-arabinose binding to GH62_2 subfamily enzymes (Siguier *et al*., 2014).

Next, we tested the Abf62C residues involved in interactions with xylotriose at all three sub-sites (Fig. 5A and B). At the +2R sub-site, respective substitution of Tyr107 or Tyr168 by alanine do not affect the specific activities in a significant way (Table 1 and Table S4). However, alanine substitution of Asn339 results in loss of activity on both arabinoxylan and pNP-α-L-arabinofuranoside (Table S4) supporting a role for this residue in substrate recognition as suggested by the structure (Fig. 5A and B). At the +1 sub-site, the individual substitution of residues Asp194, Trp229 and Tyr338 completely abrogates Abf62C enzymatic activity against wheat arabinoxylan (Table 1 and Table S4). Conversely, the D194A, W229F and W229A Abf62C variants display an obvious increase in otherwise barely detectable activity on pNP-α-L-arabinofuranoside. The replacement of Asp194 and Trp229 with smaller residues would create a larger opening around the catalytic Glu230 (Fig. 5A), which can better accommodate the p-nitrophenyl ring of pNP-α-L-arabinofuranoside. This also underlines non-catalytic roles of Asp194 and Trp229 in orienting the xylose backbone of the substrate. At the +2NR sub-site, Asp194 is also involved, together with Tyr226 in the orientation of the +2NR of xylotriose via a water molecule (Fig. 5B). This interaction is important as substitution of Tyr226 with alanine leads to complete loss of activity on wheat arabinoxylan (Table 1 and Table S4).

Overall, our mutagenesis data reveal the critical role of several Abf62C residues in the orientation of the substrate molecule in the active site. Our findings also underline the importance of remote active centre sub-sites involved in accommodation of a longer polymer backbone.

### Comparative analysis of *Abf62C* and *Abf62A*

**Fig 6:**
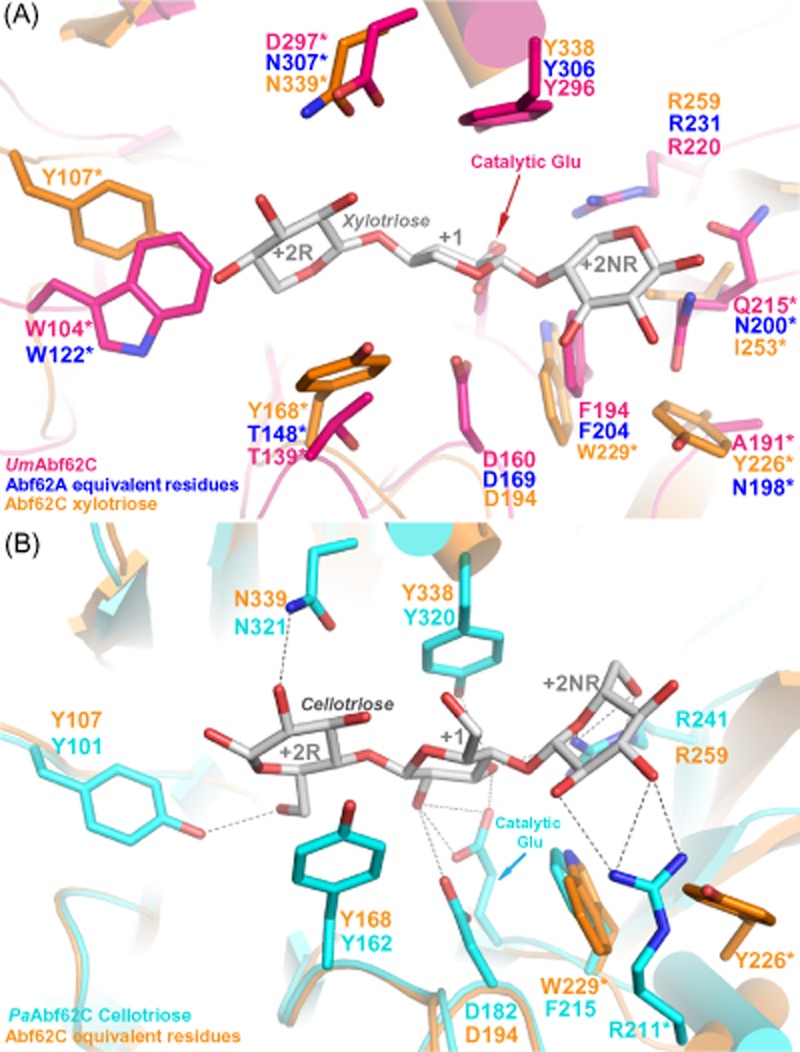
Sub-site variations between the two GH62 subfamilies.A. Overlay of *Um*Abf62C (pink) Abf62C (orange) structures. The equivalent residues of Abf62A are labelled in blue. Abf62C belongs to the GH62_1 and Abf62A and *Um*Abf62C belong to GH62_2 subfamilies. Variations in active site residues are marked with an asterisk (*).B. *Pa*Abf62C structure (cyan) in complex with cellotriose (silver grey) overlaid with Abf62C (orange). Both Abf62C and *Pa*Abf62C belong to the GH62_1 subfamily. The variations in active site residues are marked with an asterisk (*).

Abf62C and Abf62A represent two distinct subfamilies. As both *abf62A* and *abf62C* are expressed under similar conditions, we undertook a detailed comparative analysis of Abf62A and Abf62C in an attempt to identify sequence (Fig. S2) and structural features (Fig. 6A) that might distinguish the functionality between these two enzymes. Abf62C in complex with xylotriose represents the first GH62_1 subfamily structure depicting substrate interactions. A sequence alignment of various fungal GH62 sequences including Abf62A indicates high conservation of the residues that are involved in interactions with xylose and linked arabinose at the +1 sub-site (Table S3 and Fig. S2), with the exception of Trp229 of Abf62C, corresponding to Phe204 in Abf62A (Fig. 6A). However, significant variations are observed at substrate binding sub-sites +2R and +2NR (Fig. 6A and B and Fig. S4). These sub-sites in Abf62C are defined by loops connecting the α2 helix and β6 sheet, and α5 helix and β10 sheet. The corresponding loops in Abf62A and other representatives of GH62_2 subfamily are significantly shorter, suggesting an altered and unique mode of substrate binding (Fig. 6A and B and Fig. S4) for each GH62 subfamily. For example, at sub-site +2R, Abf62C residue Asn339, which is critical for substrate recognition, is often replaced by an aspartate or, to a lesser extent, with glycine. A replacement of the equivalent asparagine residue with glutamine in the case of the *Streptomyces coelicolor* enzyme (Maehara *et al*., 2014) is reported to increase its activity on longer chain substrates. Similarly, Abf62C Tyr168 appears to be conserved in members of subfamily GH62_1, whereas the equivalent position in members of the GH62_2 subfamily is occupied by threonine or alanine (Thr139 in *Um*abf62C and Thr148 in Abf62A, Fig. 6 and Fig. S2). Such a change in residue size may result in a wider cavity opening at the +2R site, thus facilitating binding of a longer or more substituted xylan backbone. Similarly, Abf62C residue Tyr226 at the +2NR sub-site is the least conserved among residues involved in the active site (Fig. 6A and B and Fig. S2). Notably, the bridged interactions between Abf62C Tyr226 and xylotriose (Fig. 5B) are compensated by non-equivalent direct hydrogen bonds between *Pa*Abf62C Arg216 and cellotriose (Fig. 6A). Further, *Um*Abf62C and Abf62A feature alanine or asparagine residues, respectively, at the position equivalent to Tyr226 in Abf62C (Fig. 6B). These alternative residues of the GH62_2 subfamily have short side chains and therefore may not be able to interact with xylotriose or cellotriose chains due to their shorter side chains, and instead might contribute towards binding an additional xylose ring at +2NR ends.

Another distinguishing feature of Abf62C is that the position equivalent to a potential calcium-binding glutamine residue of Abf62A (Gln207) ligand is replaced by a cysteine residue (Cys233; Fig. 4C and D and Fig. S2) although calcium-binding histidine residues are conserved in the two structures (His248 and His303) respectively. To probe individual roles of these residues in Abf62C and Abf62A catalytic activity, a series of mutations was designed (Table S4), and the variant enzymes were tested for activities on pNP-α-L-arabinofuranoside and wheat arabinoxylan substrates (Table S4). Substitution of the conserved histidine (His303 or His248) residues with alanine in both Abf62C and Abf62A resulted in complete inactivation. The activities of Abf62A Q207A and Q207C variants, and the C233Q variant of Abf62C, were also dramatically abrogated compared with the wild type, highlighting potential functional significance of these residues. However, the loss of activity in the Abf62C C233Q variant can be also attributed to steric clashes between the introduced glutamine's side chain and those of Glu306 and Trp174, both of which are conserved in representatives of GH62_1 subfamily (Fig. S2). The equivalent positions in Abf62A are occupied by smaller residues, Asp238 and Thr154, which are conserved in representatives of GH62_2 subfamily.

Combined with the structural analysis, our mutagenesis studies suggest that the primary role of the Abf62C His303 residue is critical for catalysis but it may not involve the coordination of metal ion, unlike equivalent residues in calcium containing GH62 enzymes.

## Discussion

Genes encoding a variety of GH43, GH51 and GH62 arabinofuranosidases are present in the genomes of many species of bacteria and fungi. These enzymes play important accessory roles in degrading arabinose-rich arabinan and arabinoxylan. The genome of the thermophilic fungus, *S. thermophilum,* features genes encoding two GH43, one GH51 and three GH62 enzymes (*abf62A*, *abf62B* and *abf62C*) (http://fungalgenomics.ca/) with potential arabinofuranosidase activities. Focussing on the GH62 family, we found that Abf62A and Abf62C were both expressed when the fungus was grown on a variety of complex substrates rich in arabinoxylan. These two enzymes represent the two subfamilies of GH62 enzymes, a combination of which is often found in fungal genomes containing multiple GH62 representatives.

To understand the significance of the coexpression of two *S. thermophilum* GH62 enzymes, we characterized their activities and obtained the crystal structure for Abf62C. During preparation of this manuscript, two other fungal GH62 enzymes, *Pa*Abf62C from *Podospora anserina* and *Um*Abf62C from *Ustilago maydis* (Siguier *et al*., 2014), and one bacterial GH62 from *S. coelicolor* (Maehara *et al*., 2014) were also structurally characterized. The GH62 structures from *U. maydis* (Siguier *et al*., 2014) and *S. coelicolor* (PDB id 3WMY, Maehara, *et al*., 2014) represents the GH62_2 subfamily, whereas the *Pa*Abf62C enzyme structure from the GH62_1 subfamily was obtained in complex with cellotriose inhibitor. We were able to determine the structure of Abf62C in complex with a true substrate component, xylotriose, thus revealing for the first time the molecular framework involved in GH62 interactions with part of a substrate molecule.

A sequence and structural comparison of Abf62C with other GH62 enzymes highlighted the conservation of residues involved in positioning of the substrate arabinose moiety. Specifically, the residues proximal to the substrate's scissile bond are invariably conserved between both GH62 subfamilies, with Abf62C showing a unique feature of Trp229 taking the place of an otherwise highly conserved phenylalanine residue. On the other hand, the residues participating in xylose binding at sub-sites +2R and +2NR show more variability that may reflect adaptation among GH62 enzymes to the diverse nature of xylan substrates.

Calcium binding is another important aspect of GH62 enzyme active sites. Several determined structures for GH43 and GH62 enzymes contain calcium ion in their catalytic pockets, anchored by a conserved histidine residue and a network of ordered water molecules. Some of the Ca^2+^ ion containing GH43 arabinanases, including BsArb43b (de Sanctis *et al*., 2010) and TpABN from *Thermotoga petrophila* (Santos *et al*., 2014) are strongly inhibited by the presence of a chelating agent. In a recently proposed mechanism, the positively charged Ca^2+^ ion in the active site of these GH43 enzymes induces hyper-polarization of an adjacent histidine residue, which in turn affects the functional protonation states of the vicinal catalytic acid residues (Santos *et al*., 2014). However, the active site of another GH43 family representative, ARN2 features a sodium ion interacting with the corresponding histidine residue (Santos *et al*., 2014) rather than a calcium ion. In the case of this enzyme, the global change in the protein architecture was proposed to contribute to the retention of the histidine molecular rotameric conformation, thus sustaining its role in catalysis (Santos *et al*., 2014). Notably, GH43 arabinanases structures lacking Ca^2+^ are not inhibited by chelation (Santos *et al*., 2014).

According to our data, the Abf62C enzyme is not affected by the presence of chelators, and no metal ion is observed in the enzyme active site despite the presence of the conserved histidine (His303) residue. These findings place Abf62C in the same category as the GH43 arabinases mentioned above (Santos *et al*., 2014) in which the proper positioning of the substrate, and catalysis, is apparently dependent on conformational flexibility of the active site residues rather than on the presence of a metal ion. Thus, we suggest that in the case of Abf62C, the active site residues, including His303, are evolved to possess the necessary flexibility to achieve a catalytically active state in the absence of a metal ion. However, at high concentrations, the large-radius divalent cations, such as Zn^2+^ and Cu^2+^, were observed to inhibit Abf62C activity, possibly through low affinity binding to the conserved histidine residue of the active site and altering the catalytic environment. Thus our data indicate that, similar to GH43 family proteins, the GH62 enzymes also demonstrate significant variation with respect to the involvement of the metal ions in the enzyme catalytic center.

The transcription profiles showed that *S. thermophilum abf62C* and *abf62A* are upregulated when cultured in plant-derived biomass, as compared with simple sugars. The coexpression of *abf62C* and *abf62A*, representing two phylogenetically distinct subtypes, under different growth conditions suggests that the concerted action of Abf62A and Abf62C enzymes provides *S. thermophilum* an edge in its natural habitat in decomposing plant-derived biomass.

In conclusion, GH62 enzymes play a prominent role in removing arabinose substituents from arabinoxylan, and thus decreasing the complexity of biomass substrates for further downstream processing. With the exponential growth of genomic data revealing a plethora of lignocellulolytic enzyme sequences, the challenge is to understand their synergetic and individual functions in the degradation of complex substrates. Microorganisms from thermophilic environments represent particularly attractive ecological niche of enzymes that can potentially carry out complete degradation of complex substrates in an industrial setting. In this respect, our data show that the GH62 family includes structurally diverse representatives that may offer unique biochemical properties that may be suitable for such applications.

## Experimental procedures

### Transcriptome analysis

*Scytalidium thermophilum* was cultured on different substrates as described (Berka *et al*., 2011). Total RNA was extracted from mycelia (Semova *et al*., 2006) at early growth phase, and sequencing was performed using the mRNA-Seq method of Illumina's Solexa IG at the McGill University-Génome Québec Innovation Centre. The RNA-Seq reads, 50 nucleotides in length, were mapped and analysed as described (Berka *et al*., 2011). Fragments per kilobase of transcript per million (mapped reads) values were calculated from the counts using the transcript lengths and the total number of mapped reads from each sample.

### DNA manipulation, cloning and expression of *abf62C* and *abf62A* in *E. coli*

Complementary DNA of *S. thermophilum* was prepared as described (Semova *et al*., 2006). Deoxyribonucleic acid fragments containing coding sequences for functional domains of Abf62A and Abf62C were amplified from double-stranded cDNA and cloned (Table S2) into an N-terminal histidine tag containing ligation independent cloning (LIC) based pET15b vector (Novagen). The cloned *abf62C* (aa30-aa350), *abf62A* (aa18-aa391), *abf62A-ΔCBM* (aa18-aa322) were expressed and purified (details in supplemental Appendix S1 Experimental procedures) from BL-21 cells (DE3) Gold strain (Stratagene). The oligonucleotide primers used for mutagenesis were designed (Table S2) using the online QuikChange Primer Design tool from Agilent Technologies and the Stratagene XL protocol.

### Activity assays

The optimal pH conditions for enzymatic activities were determined using 50 mM Britton-Robinson (BR) buffer in pH ranges 2.0–9.0 at 40°C. The same buffer at pH 6.0 was used to determine the optimal temperature using wheat arabinoxylan as substrate. Enzymatic reaction using as substrate pNP-α-L-arabinofuranoside (Sigma N3641) as substrate (1 mM substrate, 1 μg of protein in 50 μl reaction) were carried out at 40°C for 30 min in 50 mM BR buffer, pH 6.0. Reactions were terminated by the addition of 50 μl of 1M Na_2_CO_3_, and p-nitrophenol (pNP) release was determined at 410 nm. One unit of enzyme activity is defined as the amount of enzyme that releases 1 μmol of pNP per min from pNP-α-L-arabinofuranoside under these conditions. Specific activities of Abf62A, Abf62AΔCBM and Abf62C were determined by measuring the release of reducing sugars using the Nelson-Somogyi method (Green *et al*., 1989) adapted to 96 well polymerase chain reaction plates using wheat arabinoxylan (high viscosity, P-WAXYH), sugar beet arabinan (P-ARAB) and CM-Linear 1,5-α-L-arabinan (Megazyme, P-CMLA). Reaction conditions used to determine kinetic parameters are indicated in Fig. S1A, and the calculations were carried using the Michaelis–Menten equation integrated into GraphPad Prism 5.0 (GraphPad Software, USA). One unit (U) of enzyme activity is defined as the amount of enzyme required to produce 1 μmol of product/min at 50°C at optimum pH. The effect of divalent cation (CaCl_2_, NiCl_2_, ZnCl_2_, MgCl_2_, CuCl_2_, CoCl_2_; each at 0.2M concentration) supplementation or chelator (0.2 M EDTA and 0.2 M EGTA) on enzymatic activities of the GH62 enzymes were assessed in 100 mM HEPES (N-2-hydroxyethylpiperazine-N-2-ethane sulfonic acid) buffer (pH 7.0) at 50°C for 30 min using 0.2% wheat arabinoxylan.

### ^1^H-NMR assay and product analysis

^1^H-NMR experiments were carried out according to the methods described by (Sakamoto *et al*., 2011). The details of the ^1^H-NMR and HPAEC-PAD detection of released arabinose are discussed in supplemental Appendix S1 Experimental procedures.

### Sequence alignment and phylogenetic analysis

ClustalW2 (Goujon *et al*., 2010) was used to carry out the multiple protein sequence alignment as well as to calculate the phylogenetic tree. Espript (Gouet *et al*., 1999) and Figtree (http://tree.bio.ed.ac.uk/software/figtree/) were used to visualize the sequence alignment and calculated tree respectively. The sequences used in alignments were obtained from National Center for Biotechnology Information and Joint Genome Institute genomic data websites.

### Crystallization

Selenomethionine crystals of Abf62C were obtained by hanging drop method at 22°C, using 1 μl of 17 mg ml^−1^ of Abf62C within the reservoir buffer solution (0.1 M Tris pH 8.5, 2.2 M KH_2_PO_4_, 12% glycerol). Abf62C co-crystals with xylotriose were obtained by pre-incubating native Abf62C (17 mg ml^−1^) with xylotriose (2 mM at 4°C, overnight) by hanging drop method at 22°C in reservoir buffer (0.2 M KH_2_PO_4_, 20% PEG3350, 20 mM xylotriose) solution. Twenty per cent Paratone-N was supplied to reservoir solution prior to flash freezing crystals into liquid nitrogen.

### Data collection and structure determination

Crystallographic data of apo and xylotriose-incubated Abf62C were collected at the 19-ID beamline of the Structural Biology Center at the Advanced Photon Source (Argonne National Laboratory, Argonne, IL, USA) (Rosenbaum *et al*., 2006). Data were collected at a wavelength of 0.9794 Å, from the single crystals and were processed using HKL3000 (Minor *et al*., 2006). Data collection statistics are presented in Table 2. The structure of apo-Abf62C was determined using diffraction data obtained from a single (SeMet-labeled) crystal by the SAD method (Hendrickson, 1991). The hexagonal crystal contains one monomer of apo-Abf62C in the asymmetric unit. The SAD phasing, density modification and initial protein model building was accomplished in the HKL-3000 (Minor *et al*., 2006) software package integrated with shelxd, shelxe (Sheldrick, 2010), mlphare (Otwinowski, 1991), dm (Cowtan, 1994), arp/warp (Langer *et al*., 2008), solve (Terwilliger and Berendzen, 1999) and resolve (Terwilliger, 2000). The structure of Abf62C with xylotriose was determined by molecular replacement using the structure of apo-Abf62C as a search model. Molecular replacement searches were performed using the molrep program of the ccp4 suite (Vagin and Teplyakov, 1999). Both models were rebuilt using the program coot (Emsley *et al*., 2010) and refined with Phenix (Adams *et al*., 2010) and refmac 5.5 (Murshudov *et al*., 1997). The translation/libration/screw (TLS) operators were automatically determined using the program Phenix and added in the final round of the refinement. The final refinement statistics for all structures are presented in Table 2. Prior to deposition of the structure in the PDB, the quality of the structure was verified with the set of validation tools in the program coot (Emsley *et al*., 2010), as well as procheck (Laskowski *et al*., 1993) and Molprobity (Lovell *et al*., 2003). Crystal packing analysis using pisa (Krissinel and Henrick, 2007) showed limited contacts between symmetry-related molecules, strongly suggesting that Abf62C monomer (the asymmetric unit content) represents a biologically relevant unit. Electrostatic potential surfaces were calculated using the apbs PyMOL plugin (Petrey and Honig, 2003).

### PDB accession codes

The atomic coordinates of apo-Abf62C and Abf62C-xylotriose complex have been deposited in the Research Co-laboratory for Structural Bioinformatics Protein Data Bank under accession codes 4PVA and 4PVI respectively.

## Supporting Information

**Fig. S1.** Biochemistry of GH62 enzymes. A. Kinetics parameters of three GH62 enzymes of *S. thermophilum* on wheat arabinoxylan. Varying concentrations of wheat arabinoxylan (P-WAXYH) were used to determine the kinetics of Abf62C (0.5 μg of protein, 100 mM HEPES pH 7.0), Abf62A (0.5 μg of protein, 100 mM citrate buffer pH 5.0) and Abf62AΔCBM (0.5 μg of protein, 100 mM citrate buffer pH 5.0) at 50°C for 30 min. B. ^1^H-NMR. ^1^H-NMR spectra of untreated (A–C) and pretreated with AFase (D–F) wheat arabinoxylan (P-WAXYL). Peaks are labelled based on assignments by Sakamoto *et al*. 2011 as follows: (1, 4) an arabinose residue bound to C-3 of a single-substituted xylose residue (5.357 ppm), (2) an arabinose residue bound to C-3 of a double-substituted xylose residue (5.240 ppm), (3) an arabinose residue bound to C-2 of a double-substituted xylose residue (5.188 ppm) and (5) an arabinose residue bound to C-2 of a single-substituted xylose residue (5.250 ppm). (A) substrate only, (B) Abf62C, (C) Abf62A, (D) pretreated substrate only, (E) Abf62C and (F) Abf62A. All spectra were recorded on a Varian VNMRS-500 MHz spectrometer at 30°C. C. HPAEC-PAD. Product analysis of Abf62C (3.4 μg ml^−1^) activity on wheat arabinoxylan (P-WAXYL; 0.2%) in Britton-Robinson buffer pH 5 (30 mM) at 40°C for 30 min. (A) A mixture of 0.016 mM arabinose and 0.016 mM xylose as standards. (B) Only arabinose is detected in the enzymatic reaction of Abf62C and arabinoxylan. Monosaccharides were detected using a Dionex ICS-500 HPLC equipped with a Carbopac PA20 analytical column (3 mm × 150 mm). **Fig. S2.** Arabinose binding in Abf62C. A. H-bonding network formed by the central phosphate in the active site of the apo Abf62C structure. B. Arabinose binding interactions of *Um*Abf62C (magenta) and their equivalent residues in Abf62C (orange). **Fig. S3.** Molecular surface of Abf62C. A. Molecular surface of xylotriose bound Abf62C (grey). The active site residues lining the binding pocket are shown in sticks (orange). B. Electrostatic surface of Abf62C displaying the xylotriose bound by a highly positively charged (red) surface extending from the catalytic core. Red indicates negative potential, white is neutral, blue shows positive potential and surfaces were contoured between −20 and +20 kB T/e, where kB is the Boltzmann constant, T is temperature and e is the electronic charge. **Fig. S4.** Protein sequence alignment between the GH62 enzymes of selected fungi. The secondary structure of Abf62C (Subfamily 1) and *Um*Abf62C residues (Subfamily 2) are presented on the top and bottom of the alignment respectively. The two GH62 subfamilies and the key residues involved in active centre of Abf62C are marked. The alignment figure was prepared by Espript (http://espript.ibcp.fr/ESPript/ESPript). **Table S1.** Sequence and structural homologies between GH62 enzymes. **Table S2.** Primers sequences used to amplify and mutate target DNA. **Table S3.** H-bonds and stacking interactions between Abf62C protein residues with sugars of xylotriose. At sub-site +2R, Abf62C residue Tyr107 forms stacking interactions with the plane of the +2R xylose of xylotriose, while side chains of Asn338 form three hydrogen bonds with two hydroxyl groups of the sugar. The arrangement/location of the xylose ring at the +1 sub-site is imperative for the catalytic Glu230 of Abf62C to access the scissile bond. Furthermore, the +1 xylose ring is oriented at the sub-site by stacking with the aromatic side chain of Tyr339 and by forming multiple hydrogen bonds with active site residues, including side chains of the catalytic Glu230 (one to 2-OH and two to 3-OH), Trp229 (2-OH), Arg259 (2-OH) and Asp194 (3-OH). The hydroxyl groups (2-OH and 3-OH) of the +2NR xylose ring form hydrogen bonds with two water molecules, which are in turn oriented by interactions with Tyr226 and Asp194. **Table S4.** Summary of site directed mutants of Abf62C and Abf62A. **Appendix S1.** Experimental procedures.
