# Supplementary material for: Functional and structural diversity in GH62 α-L-arabinofuranosidases from the thermophilic fungus Scytalidium thermophilum
Source: Microb Biotechnol. 2014 Sep 29;8(3):419–33. doi: 10.1111/1751-7915.12168 (PMC4408175; doi:10.1111/1751-7915.12168)
Supplement: Supplementary file 1 [file mbt20008-0419-sd1.zip › mbt212168-sup-0001-tablesS1-S4.doc.docx]

**SUPPLEMENTAL DATA**

**Supplementary Experimental Procedures**

**Protein purifications.** Abf62C and Abf62A proteins and their variants were purified from BL-21 cells after 16 hours of growth at 18^°^C (Abf62C) or 16°C for (Abf62A, Abf62AΔCBM, after inducing with 1 mM IPTG at A_600_= 0.7). Cultured *E. coli* cells were pelleted by centrifugation at 8000 rpm and lysed by ultra-sonication in buffer A (50 mM HEPES, 300 mM NaCl, 5 mM imidazole, 5% glycerol). Histidine-tagged recombinant proteins from cell lysates were purified by nickel resin (Qiagen, Ni-NTA) chromatography using the manufacturer’s protocol. Buffer B (50 mM HEPES pH 7.5, 500 mM NaCl, 30 mM imidazole, 5% glycerol) was used to wash the nickel column and Buffer C (50 mM HEPES pH 7.5, 500 mM NaCl, 300 mM imidazole, and 5% glycerol) was used to elute the protein. The purified proteins were dialyzed (dialysis buffer composition was 10 mM HEPES pH 7.5, 500 mM NaCl, 5% glycerol) overnight using snakeskin dialysis tubing (M.W.C.O 10 kDa), and then concentrated for crystallization purposes.

**Product Analysis.** Reaction mixtures (50 µL) consisting of of 1% wheat arabinoxylan (Megazyme, P-WAXYL), 50 mM Britton-Robinson buffer pH 5 (50 mM boric acid, 50 mM acetic acid, and 50 mM phosphoric acid) and Abf62C (17 µg/ml) were incubated at 40 °C for 30 minutes. The reactions were stopped by heating at 95 °C for 5 minutes, diluted 20-fold in water, and analyzed by high performance anionic exchange chromatography with pulsed amperometric detection (HPAEC-PAD) with an ICS-5000 HPLC system (Dionex, USA). Analyses of standards and samples (10 µL injections) were conducted at 30 °C with isocratic elution in 100 mM NaOH (0.5 mL/min) using a Carbopac PA20 analytical column (3 mm x 150 mm) and a guard (3 mm x 30 mm). Eluted samples were monitored with a gold electrode on PTFE and all potentials were reported versus an Ag/AgCl electrode. A mixture of L-arabinose (Sigma, A3256) and D-xylose (BioShop, XYL001) were prepared at a concentration of 0.016 mM each in water and used as a standard.

**^1^H-NMR assay.** ^1^H-NMR experiments were carried out according to the methods described by Sakamoto *et* al, 2011. Briefly, a 50-mL solution of 0.2% wheat arabinoxylan (Megazyme, P-WAXYL) was prepared in 20 mM potassium phosphate buffer (pH 6.0). A total of 10 U of α-L-arabinofuranosidase AFase (Megazyme, E-AFAM2) from *Bifiobacterium* sp. was added and the mixture was incubated at 37 °C for 48 hours with rotation. The mixture was boiled for 15 minutes and dialyzed against water (14 KDa MWCO dialysis tubing), lyophilized, resuspended to a concentration of 0.5% in water, and syringe filtered (0.2 µm). A control sample of wheat arabinoxylan was processed simultaneously without the addition of the AFase. Solutions of 0.1% un-treated and AFase-treated wheat arabinoxylan were prepared in 50 mM sodium acetate buffer (pH 5.0) and incubated with 60 milliU of Abf62A or Abf62C for 16 hours at 37° C with rotation. The samples were boiled for 15 minutes, dialyzed against water (14KDa MWCO dialysis tubing), lyophilized, resuspended with 500 µL of 99.9% deuterium oxide (D_2_O) (Sigma-Aldrich, 151882), centrifuged at 4200 x g for 10 minutes, transferred to 1.5 mL tubes, and centrifuged at 16,000 x g for 10 minutes. After transferring the supernatant to a new tube, the samples were lyophilized and re-suspended in 600 µL of D_2_O. ^1^H-NMR spectra were recorded on a Varian VNMRS-500 MHz spectrophotometer at 30°C.

**SUPPLEMENTARY TABLES**

**Table S1**: Sequence and structural homologies between GH62 enzymes.

| Table S1 | Sequence/structure homologies | | | | | |
| --- | --- | --- | --- | --- | --- | --- |
| Protein | **Abf62A** | **Abf62B** | ***Um*Abf62C** | | ***Pa*Abf62C** | |
|  | % identity | % identity | % identity | R.M.S.D.  (Å) | % identity | R.M.S.D.  (Å) |
| Abf62C | 32 | 34 | 34 | 0.82 | 64 | 0.23 |
| Abf62A |  | 60 | 53 | - | 30 | - |
| *Um*Abf62C |  |  |  |  | 32 | 0.82 |

**Table S2:** Primers sequences used to amplify and mutate target DNA.

| Table S2 | Primer sequences |
| --- | --- |
| Cloning primers |  |
| Abf62A_18_fr | 5’-TTGTATTTCCAGGGCGCGTGCAGTCTCCCATCC-3’ |
| Abf62A_322_rv | 5’-CAAGCTTCGTCATCACCGGGCACCGACCATGG-3’ |
| Abf62A_391_rv | 5’-CAAGCTTCGTCATCACAGGCATTGAGAGTACCAAG-3’ |
| Abf62C_30_350_fr | 5’-GCGGCGGCCCATATGAGCAGCTGGAAATGGGTTTC-3’ |
| Abf62C_30_350_rv | 5’-GCGCAGATCTCTGAGTGATCAACCCCAAC-3’ |
| Mutagenesis Primers | |
| Abf62A_F203W_fr | 5’-CGCAACGACCTCTGGGAGGCCGTCCAGG-3’ |
| Abf62A_F203W_rv | 5’-CCTGGACGGCCTCCCAGAGGTCGTTGCG-3’ |
| Abf62C_W229A_fr | 5' CAGAACATCTACGCCCTCGCAGAAGCCGCGTGCATCTAC 3' |
| Abf62C_W229A_rv | 5' GTAGATGCACGCGGCTTCTGCGAGGGCGTAGATGTTCTG 3' |
| Abf62C_D55A_fr | 5'-ggcatcaaggcccctaccgccgtcctgatcaacgg-3' |
| Abf62C_D55A_rv | 5'-Acggcggtaggggccttgatgccggcgatgttgcg-3’ |
| Abf62C_D171A_fr | 5'-cggctactgggttgccatgtgggtggtctgcg-3' |
| Abf62C_D171A_rv | 5'-Agaccacccacatggcaacccagtagccgttgccg-3’ |
| Abf62C_D194A_fr | 5'- cttctcgtccgacgccaacggccatctttaccgc-3' |
| Abf62C_D194A_rv | 5'-Gatggccgttggcgtcggacgagaagaggtgg-3’ |
| Abf62C_Y77A_fr | 5'- ccaagtccgaaggcgccaacatggtgtacttcaacttc-3 |
| Abf62C_Y77A_rv | 5'-TGAAGTACACCATGTTGGCGCCTTCGGACTTGGCCG -3’ |
| Abf62C_Y226A_fr | 5'-Cacccagaacatcgccgccctctgggaagccgcg-3' |
| Abf62C_Y226A_rv | 5'-Cttcccagagggcggcgatgttctgggtgtcctgc-3’ |
| Abf62C_Y338A_fr | 5'-tgcgcagactgaggctaacgccctgccgtggagg-3’ |
| Abf62C_Y338A_rv | 5'-Acggcagggcgttagcctcagtctgcgcattagg-3’ |
| Abf62C_W229A_fr | 5-catctacgccctcgcggaagccgcgtgcatc-3' |
| Abf62C_W229A_rv | 5'-Gcacgcggcttccgcgagggcgtagatgttctgg-3’ |
| Abf62C_ H303A_ fr | 5'- ccaagagcatcagcgccggcgaagtgatcaggacc-3’ |
| Abf62C_H303A_ rv | 5'-Cctgatcacttcgccggcgctgatgctcttggtc-3’ |
| Abf62C_E230A_fr | 5'-cgccctctgggcagccgcgtgcatcaccgc-3' |
| Abf62C_E230A_rv | 5’-Tgcacgcggctgcccagagggcggcg-3’ |
| Abf62C_Y107A_rv | 5’-GAGCCGCGCGGGCGCCGTAGCCGAGAG-3’ |
| Abf62C_Y107A_fr | 5’-CTCGGCTACGGCGCCCGCGCGGCTCCGCAAG-3’ |
| Abf62C_K54A_fr | 5’-GCGGTAGGGTCCGCGATGCCGGCGATGTTGC-3’ |
| Abf62C_K54A_rv | 5’-CATCGCCGGCATCGCGGACCCTACCGCCGTC-3’ |
| Abf62C_N339A_fr | 5’-TGCGCAGACTGAGTATGCCGCCCTGCCGTGGAGG-3’ |
| Abf62C_N339A_rv | 5’-ACGGCAGGGCGGCATACTCAGTCTGCGCATTAGG-3’ |
| Abf62C_Y168A_rv | 5’-CCACATGTCAACCCAGGCGCCGTTGCCGATCTTG-3’ |
| Abf62C_Y168A_fr | 5’-CAAGATCGGCAACGGCGCCTGGGTTGACATGTGG-3’ |
| Abf62C_W230F_fr | 5’-ACATCTACGCCCTCTTCGAAGCCGCGTGCATC-3’ |
| Abf62C W230F_rv | 5’-GATGCACGCGGCTTCGAAGAGGGCGTAGATGT-3’ |
| Abf62C_R2590A_fr | 5’-GGCCAGGAAGGTCACGCGTACTTCCGCTCGTG-3’ |
| Abf62C_R259A_rv | 5’-CACGAGCGGAAGTACGCGTGACCTTCCTGGCC-3’ |
| Abf62A_Q207A_fr | 5’-CCTCTTCGAGGCCGTCGCGGTCTACACCATTGAC-3’ |
| Abf62A_Q207A_rv | 5’-GTCAATGGTGTAGACCGCGACGGCCTCGAAGAGG-3’ |
| Abf62A_Q207C_fr | 5’-ACCTCTTCGAGGCCGTCTGCGTCTACACCATTGACGG-3’ |
| Abf62A_Q207C_rv | 5’-CCGTCAATGGTGTAGACGCAGACGGCCTCGAAGAGGT-3’ |
| Abf62A_H234A_fr | 5’-TCCGACATCAGCGCAGGCGACCTCGTCCGC-3’ |
| Abf62A_H234A_rv | 5’-GACGAGGTCGCCTGCGCTGATGTCGGAGGT-3’ |
| Abf62C_Y168T_rv | 5’-CCACATGTCAACCCAGGTGCCGTTGCCGATGTTG-3’ |
| Abf62C_Y168T_fr | 5’-CAACATCGGCAACGGCACCTGGGTTGACATGTGG-3’ |
| Abf62C_C233Q_rv | 5’-CCTTGATGCGGTAGATCTGCGCGGCTTCCCAGAGG-3’ |
| Abf62C_C233Q_fr | 5’-CCTCTGGGAAGCCGCGCAGATCTACCGCATCAAGG-3’ |
| Abf62C_Y168A_fr | 5’-CAACATCGGCAACGGCGCCTGGGTTGACATGTGG-3’ |
| Abf62C_Y168A_rv | 5’-CCACATGTCAACCCAGGCGCCGTTGCCGATGTTG-3’ |
| Abf62C_Y226R_fr | 5’-GACACCCAGAACATCCGCGCCCTCTGGGAAGC-3’ |
| Abf62C_Y226R_rv | 5’-GCTTCCCAGAGGGCGCGGATGTTCTGGGTGTC-3’ |

**Table S3**: **H-bonds and stacking interactions between Abf62C protein residues with sugars of xylotriose.** At subsite +2R, Abf62C residue Tyr107 forms stacking interactions with the plane of the +2R xylose of xylotriose, while side chains of Asn338 form three hydrogen bonds with two hydroxyl groups of the sugar. The arrangement/location of the xylose ring at the +1 subsite is imperative for the catalytic Glu230 of Abf62C to access the scissile bond. Furthermore, the +1 xylose ring is oriented at the subsite by stacking with the aromatic side chain of Tyr339 and by forming multiple hydrogen bonds with active site residues, including side chains of the catalytic Glu230 (one to 2-OH and two to 3-OH), Trp229 (2-OH), Arg259 (2-OH) and Asp194 (3-OH). The hydroxyl groups (2-OH and 3-OH) of the +2NR xylose ring form hydrogen bonds with two water molecules, which are in turn oriented by interactions with Tyr226 and Asp194.

| Table S3 | Interactions between Abf62C and xylotriose | | |
| --- | --- | --- | --- |
| Subsite | **Substrate atom** | **Protein atom** | **Distance (Å)** |
| +2R | OH-2 | Asn339 N^δ1^ | 2.7 |
|  | OH-2 | Asn339 N^δ2^ | 3.2 |
|  | OH-3 | Asn339 N^δ2^ | 3.1 |
|  |  | Tyr168 | hydrophobic stacking |
| +1 | OH-2 | Glu230 O^€1^ | 2.7 |
|  | OH-3 | Glu230 O^€2^ | 3.4 |
|  | OH-3 | Glu230 O^€2^ | 2.7 |
|  | OH-2 | Trp229 N^€1^ | 3.1 |
|  | OH-2 | Asp194 O^δ2^ | 3.5 |
|  | OH-2 | Arg259 N^η2^ | 2.9 |
|  |  | Tyr338 | hydrophobic stacking |
| +2NR | OH-2 | Tyr226 O^η1^ | Linked through a single water molecule. |
|  | OH-3 | Tyr226 O^η1^ |  |

**Table S4**: Summary of site directed mutants of Abf62C and Abf62A.

| Table S4 | Selected residues | Activity on arabinoxylan | Activity on pNP-arabinofurnanoside |
| --- | --- | --- | --- |
| Abf62C | **Wild type** | +++ | + |
| Catalytic core | ***catalytic triad*** | | |
|  | D55A | - | - |
|  | D171A | - | - |
|  | E230A | - | - |
|  | ***Arabinose recognition*** | | |
|  | K54A | - | - |
|  | Y77F | - | - |
|  | Y77A | - | - |
|  | R259A | - | - |
|  | H303A | - | - |
|  | Y338A | - | - |
| +2R | Y107A | +++ | + |
|  | Y168A | ++++ | + |
|  | Y168T | +++ | + |
|  | N339A | - | - |
| +1 | D194A | - | - |
|  | W229A | - | ++ |
|  | W229F | - | ++ |
|  | Y338A | - | - |
|  | R259A | - | - |
| +2NR | Y226A | - | - |
| Calcium interaction | C233Q | - | - |
|  | H303A | - | - |
| Variants of Abf62A | Q207A | - | - |
|  | Q207C | - | - |
|  | H248A | - | - |
|  | F204W | ++++ | ++++ |

**SUPPLEMENTARY FIGURES LEGENDS**

**Fig S1. Biochemistry of GH62 enzymes.**

(A) Kinetics parameters of three GH62 enzymes of *S. thermophilum* on wheat arabinoxylan.

Varying concentrations of wheat arabinoxylan (P-WAXYH) were used to determine the kinetics of Abf62C (0.5 µg of protein, 100 mM HEPES pH 7.0), Abf62A (0.5 µg of protein, 100 mM citrate buffer pH 5.0) and Abf62AΔCBM (0.5 µg of protein, 100 mM citrate buffer pH 5.0) at 50°C for 30 minutes.

(B) ^1^H-NMR. ^1^H-NMR spectra of untreated (A-C) and pre-treated with AFase (D-F) wheat arabinoxylan (P-WAXYL). Peaks are labelled based on assignments by Sakamoto et al. 2011 as follows: (1, 4) an arabinose residue bound to C-3 of a single-substituted xylose residue (5.357 ppm), (2) an arabinose residue bound to C-3 of a double-substituted xylose residue (5.240 ppm), (3) an arabinose residue bound to C-2 of a double-substituted xylose residue (5.188 ppm), and (5) an arabinose residue bound to C-2 of a single-substituted xylose residue (5.250 ppm). (A) substrate only, (B) Abf62C, (C) Abf62A, (D) pre-treated substrate only, (E) Abf62C, and (F) Abf62A. All spectra were recorded on a Varian VNMRS-500 MHz spectrometer at 30 °C.

(C) HPAEC-PAD. Product analysis of Abf62C (3.4 µg/ml) activity on wheat arabinoxylan (P-WAXYL; 0.2%) in Britton-Robinson buffer pH 5 (30 mM) at 40 °C for 30 minutes. (A) A mixture of 0.016 mM arabinose and 0.016 mM xylose as standards. (B) Only arabinose is detected in the enzymatic reaction of Abf62C and arabinoxylan. Monosaccharides were detected using a Dionex ICS-500 HPLC equipped with a Carbopac PA20 analytical column (3 mm x 150 mm).

**Fig S2. Arabinose binding in Abf62C.**

(A) H-bonding network formed by the central phosphate in the active site of the apo Abf62C structure.

(B) Arabinose binding interactions of *Um*Abf62C (magenta) and their equivalent residues in Abf62C (orange).

**Fig S3. Molecular surface of Abf62C.**

(A) Molecular surface of xylotriose bound Abf62C (grey). The active site residues lining the binding pocket are shown in sticks (orange).

(B) Electrostatic surface of Abf62C displaying the xylotriose bound by a highly positively charged (red) surface extending from the catalytic core. Red colour indicates negative potential, white is neutral, blue shows positive potential and surfaces were contoured between -20 and +20 kB T/e, where kB is the Boltzmann constant, T is temperature, and e is the electronic charge.

**Fig S4. Protein sequence alignment between the GH62 enzymes of selected fungi.**

The secondary structure of Abf62C (Subfamily 1) and *Um*Abf62C residues (Subfamily 2) are presented on the top and bottom of the alignment, respectively. The two GH62 subfamilies and the key residues involved in active centre of Abf62C are marked. The alignment figure was prepared by Espript (http://espript.ibcp.fr/ESPript/ESPript).
