# Supplementary figures and images for: Functional and structural diversity in GH62 α-L-arabinofuranosidases from the thermophilic fungus Scytalidium thermophilum
Source: Microb Biotechnol. 2014 Sep 29;8(3):419–33. doi: 10.1111/1751-7915.12168 (PMC4408175; doi:10.1111/1751-7915.12168)

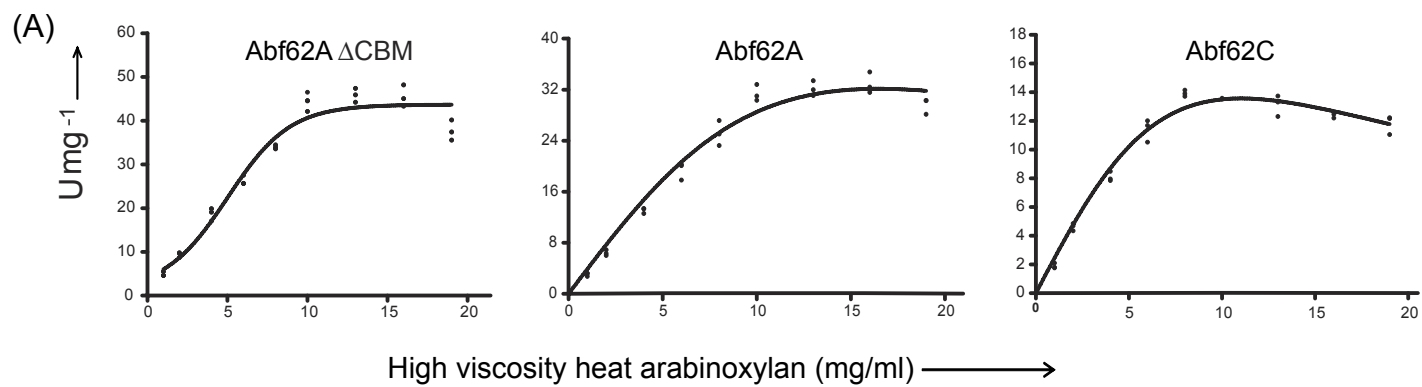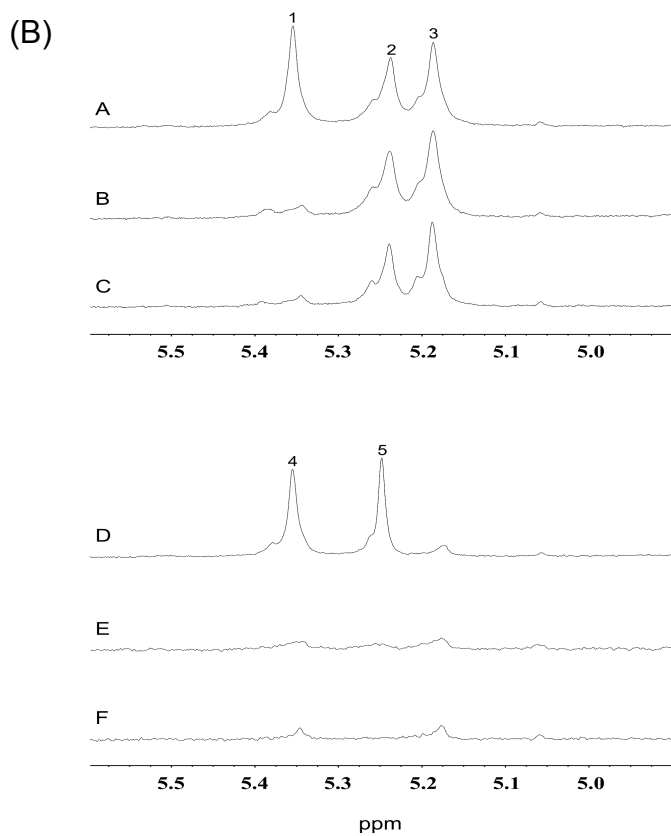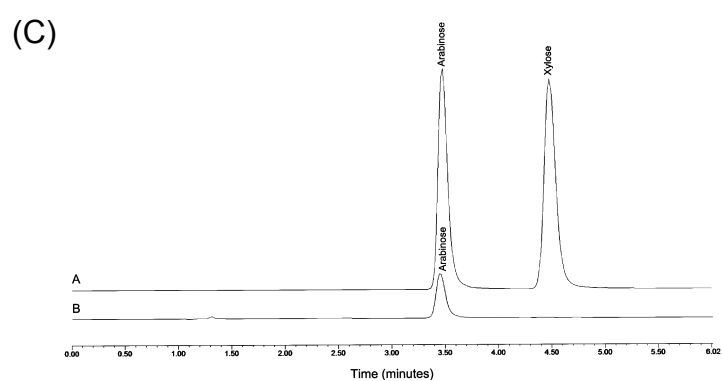

Figure S1

Supplement: Supplementary file 1 [file mbt20008-0419-sd1.zip › mbt212168-sup-0001-figureS1.pdf.pdf]

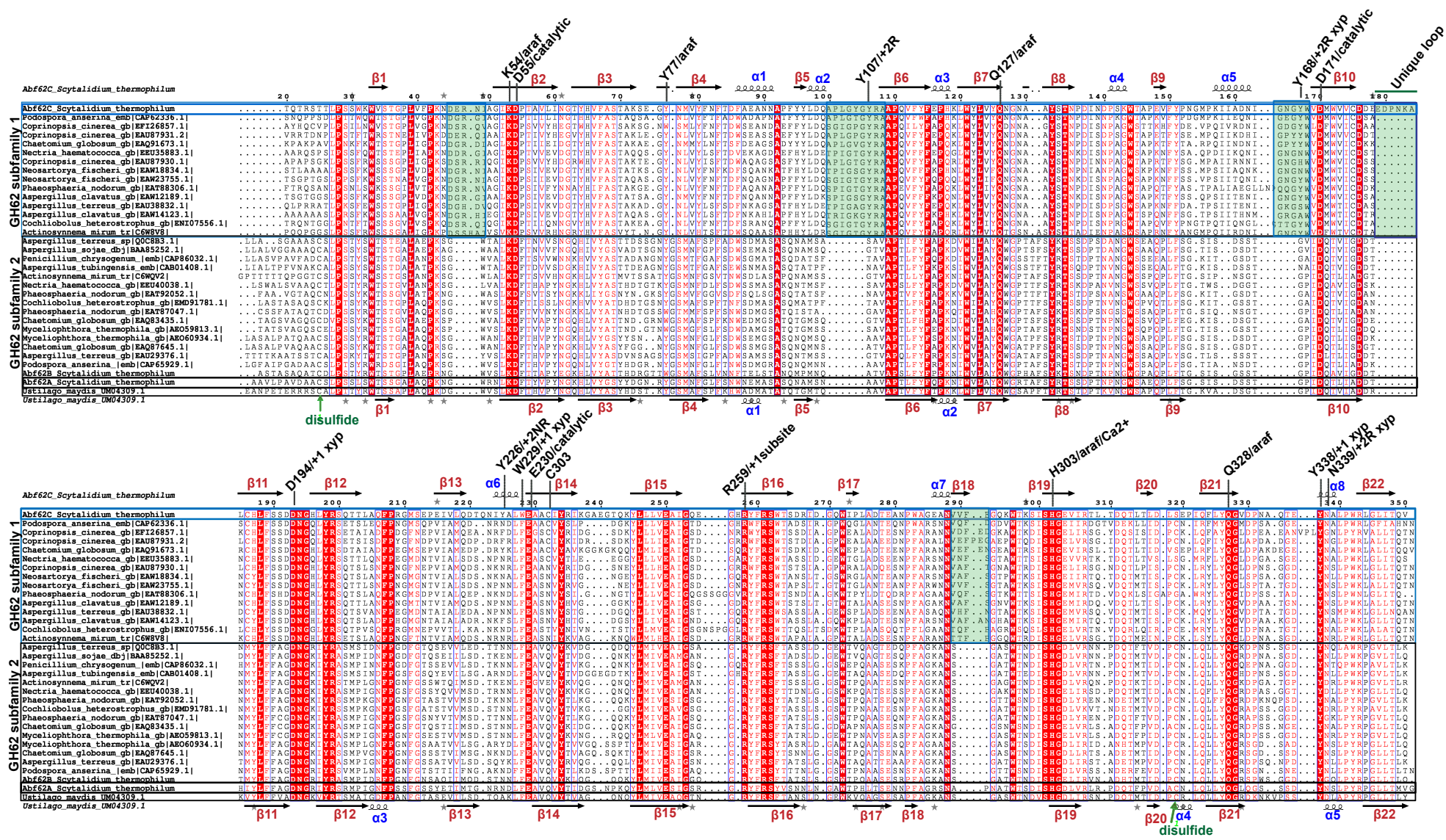

Figure S2

Supplement: Supplementary file 1 [file mbt20008-0419-sd1.zip › mbt212168-sup-0002-figureS2.pdf.pdf]

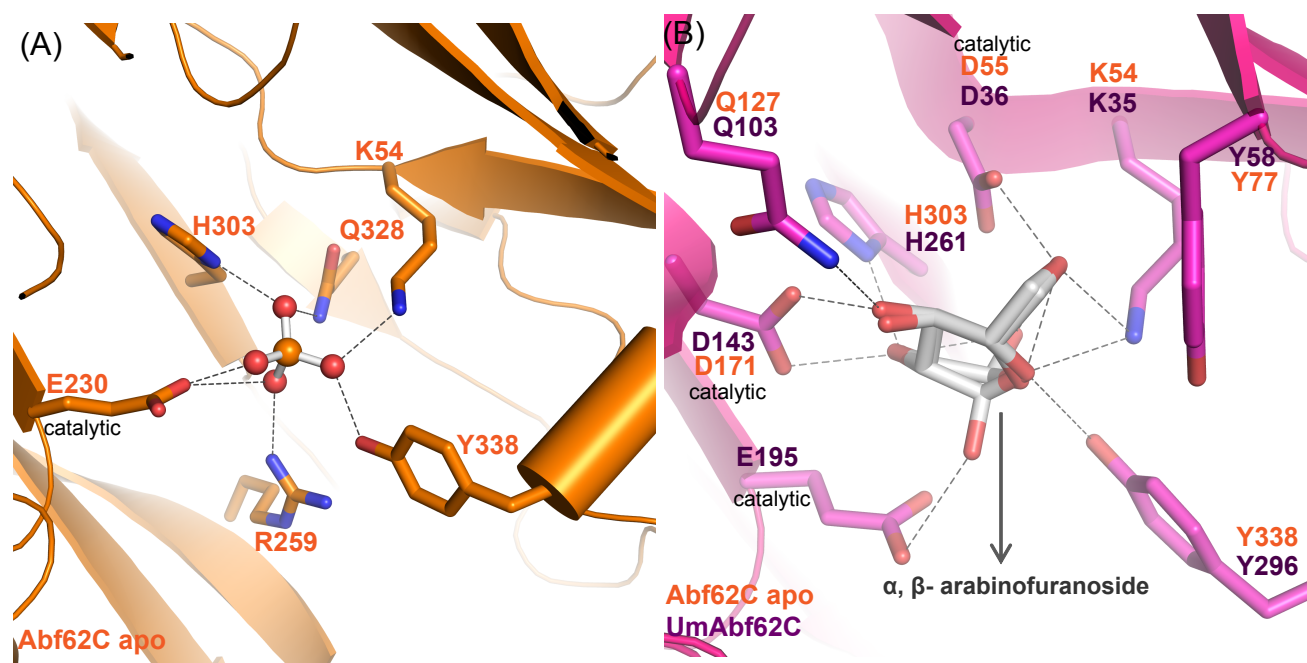

Figure S3

Supplement: Supplementary file 1 [file mbt20008-0419-sd1.zip › mbt212168-sup-0003-figureS3.pdf.pdf]

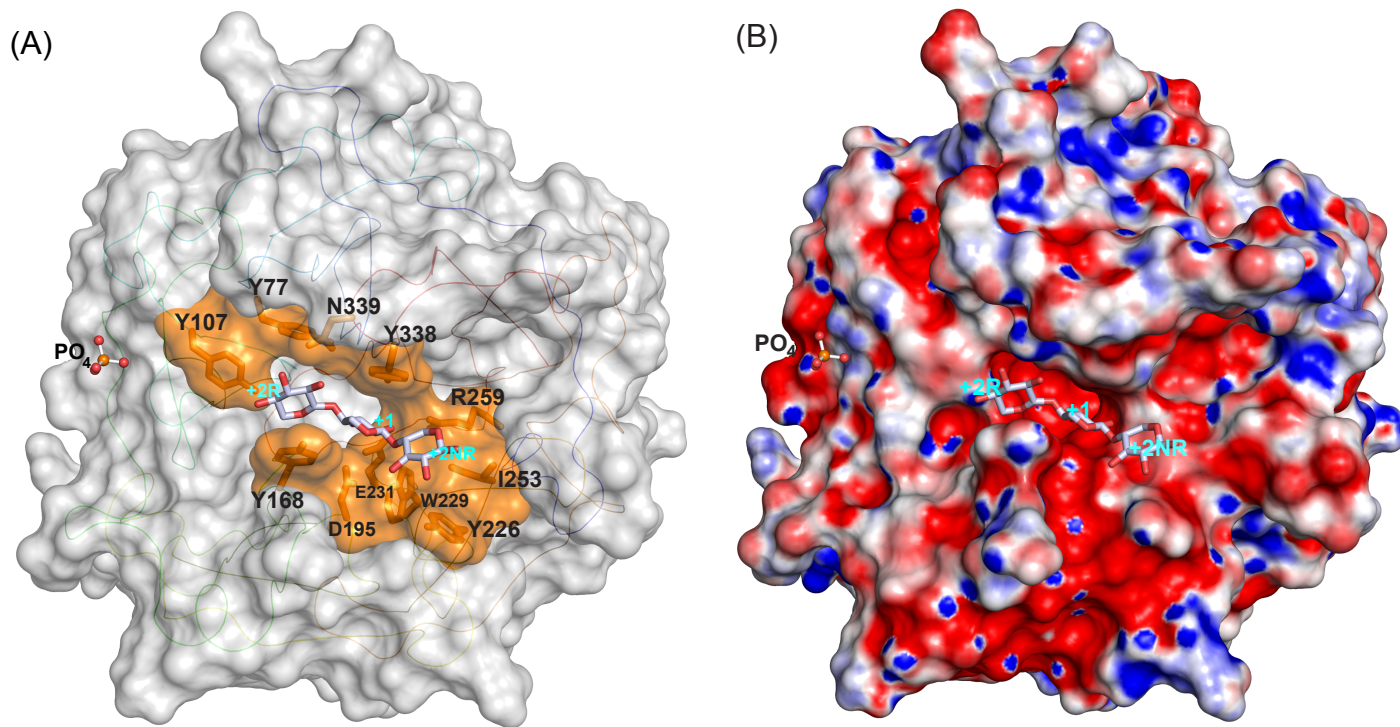

Figure S4

Supplement: Supplementary file 1 [file mbt20008-0419-sd1.zip › mbt212168-sup-0004-figureS4.pdf.pdf]
